# Again Unguentine

**Published:** 1909-10

**Authors:** Frank A. Barber

**Affiliations:** Chicago


AGAIN UNGUENTINE.—Dr. Downs' article on the use of Unguentine in a burn from steam calls to mind two cases in which I received the best of results by the use of the same article. My first experience with it was treating a wound caused by an operation for the removal of a fatty tumor over the right shoulder blade in a lady that was to a degree scrofulous. The surgeon made his incision perpendicularly over the tumor, the consequence being an impossibility to hold the parts together, the least motion causing a tearing loose. She was two years trying first one physician and then another, and from one school to another. Finally she wrote me asking me to meet her and go with her to some surgeon in Chicago. I made her the proposition that she allow me to treat the wound three weeks. If I did no good she should have her treatment for nothing, including board at my home; if I cured her, she to pay me the same fee she had others. I accordingly applied Unguentine, changing night and morning. Result, a perfect healing by granulation in two weeks, and no eschar.

My second case was that of a lady whose entire right arm ignited while cleaning a pair of suede gloves drawn on the hand and arm—a terrible burn, reaching from the tips of the fingers to the shoulder joint. Unguentine, first and last for three weeks, healed it with absolutely no pain in the interim and also no eschar.—Frank A. Barber, M. D., Chicago, in *Ellingwood's Therapeutist,* June 15, 1909.

---

BATTLE & Co., St. Louis, have just issued No. 10 of their dislocation chart series. They will send all back numbers, free of charge, to physicians on request.

